# From undruggable to degradable: A deep learning-enabled framework for precision orthopaedic protein degradation

**DOI:** 10.1016/j.jot.2026.101118

**Published:** 2026-05-11

**Authors:** Lulu Zhang, Yan Wang, Dong Wang, Jianxiong Ma

**Affiliations:** a Tianjin Hospital, Tianjin University, Tianjin, 300211, China; b Tianjin Orthopedic Institute, Tianjin, 300050, China; c Tianjin University of Traditional Chinese Medicine, Tianjin 301617, China; d Tianjin Institute of Integrated Traditional Chinese and Western Medicine Orthopedics, Tianjin 300050, China

**Keywords:** Targeted protein degradation, Deep learning, Orthopaedics, PROTACs, Molecular glues, Translational medicine

## Abstract

**Background/objective:**

A fundamental translational impasse in musculoskeletal disorders—from osteoarthritis to bone metastases—is the “undruggability” of critical pathological drivers sequestered within anatomically restricted and avascular skeletal niches. While Targeted Protein Degradation (TPD) offers a revolutionary event-driven modality to eradicate these refractory targets, its clinical translation to orthopaedics is severely hindered by the physicochemical incompatibility of large degraders with dense extracellular matrices and the scarcity of bone-specific E3 ligases.

**Methods:**

In this review, we present a transformative framework integrating deep learning (DL) with multimodal omics to circumvent these barriers. We systematically examine how emerging DL architectures—spanning geometric deep learning, protein language models, and generative design—are redefining the orthopaedic TPD pipeline.

**Results:**

We highlight computational strategies for prioritizing cryptic pockets on “undruggable” skeletal transcription factors, identifying tissue-restricted E3 ligases via single-cell transcriptomics to minimize systemic toxicity, and optimizing degrader permeability (“penetrability-first” design) to navigate dense cartilage and bone matrices.

**Conclusion:**

By bridging the gap between computational prediction and skeletal pathobiology, this roadmap outlines a shift in orthopaedic care from palliative symptom management to precise, mechanism-based microenvironmental reprogramming.

**The Translational Potential of this Article:**

This review provides a timely, computationally-driven roadmap to translate Targeted Protein Degradation (TPD) from oncology to orthopaedics. By integrating deep learning with skeletal pathobiology to overcome matrix penetrability and off-target toxicity, this framework accelerates the development of precision degraders. Ultimately, it offers a tangible strategy to shift the clinical management of joint degeneration, osteoporosis, and bone tumors from palliative care to durable, mechanism-based microenvironmental reprogramming.

## Graphical abstract

**Figure:**
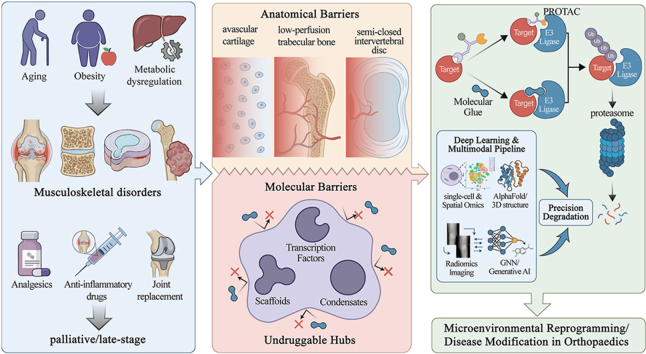

## 1 Introduction: Orthopaedic diseases, microenvironmental barriers, and the “Undruggable” challenge

**Fig. 1:**
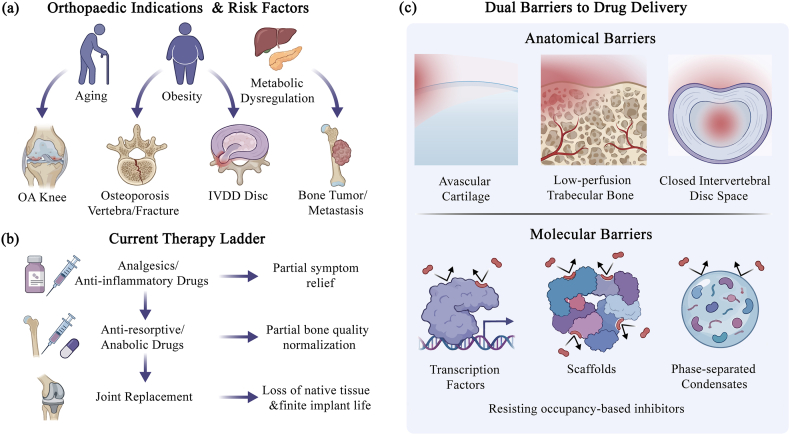
**Therapeutic ceiling in orthopaedics arises from the convergence of anatomical and molecular barriers.** (a) Representative orthopaedic indications and their major population-level drivers. Aging, obesity, and metabolic dysregulation collectively promote osteoarthritis, osteoporosis-related vertebral pathology, intervertebral disc degeneration, and bone tumors/metastases. (b) The current treatment ladder, from analgesics and broad anti-inflammatory agents to anti-resorptive/anabolic drugs and end-stage joint replacement, provides incomplete or transient benefit and often sacrifices native tissue. (c) Dual barriers that stall durable disease modification: steep transport constraints imposed by avascular cartilage, low-perfusion trabecular bone, and the semi-closed disc space, together with disease-maintaining “undruggable” signaling hubs (transcription factors, scaffolding/condensate proteins, and non-enzymatic nodes) that evade conventional inhibition.

Musculoskeletal disorders—encompassing a broad spectrum of recalcitrant pathologies ranging from osteoarthritis (OA) and osteoporosis to intervertebral disc degeneration (IVDD) and malignant bone tumors—constitute one of the most pervasive sources of global disability and socioeconomic burden [1,2]. As the global population confronts the converging challenges of aging, the obesity epidemic, and metabolic dysregulation, the prevalence of these conditions is escalating into a silent crisis(Fig. 1a) [1,2]. Despite this growing urgency, the current orthopaedic therapeutic armamentarium remains disproportionately reliant on palliative measures that fail to address the root molecular etiologies. Clinical interventions are largely restricted to symptomatic pain management, non-specific anti-inflammatory regimens, systemic modulation of bone mass, or, in end-stage disease, the salvage replacement of compromised tissues with prosthetic implants [2,3]. A critical deficiency in contemporary orthopaedics is the paucity of strategies capable of durably “reprogramming” the pathological microenvironment to restore native tissue homeostasis(Fig. 1b) [2,3]. This therapeutic ceiling is fundamentally reinforced by the unique and unforgiving anatomical architecture of the skeletal system, which functions as a formidable barrier to pharmacological intervention. The localized niches of bone and joint tissues present steep biophysical obstacles: articular cartilage is avascular, dense, and rich in negatively charged proteoglycans; trabecular bone and the intervertebral disc are poorly perfused regions characterized by high interstitial pressure; and the bone marrow cavity operates as a compartmentalized, semi-closed ecosystem [3,4]. These characteristics create unfavorable drug concentration gradients, preventing systemic agents from penetrating the dense extracellular matrix (ECM) to reach therapeutic thresholds within the disease nidus(Fig. 1c) [3,4]. Consequently, a profound disconnect frequently arises between promising preclinical laboratory indicators and the lack of tangible structural preservation or symptomatic relief observed in clinical imaging and patient outcomes.

Beyond these macroscopic anatomical barriers lies a more profound impediment at the molecular level: the intrinsic “undruggability” of the master regulators driving skeletal pathogenesis [5,6]. From the perspective of conventional medicinal chemistry, the core nodes of the signalling networks governing bone and joint deterioration are largely inaccessible. Traditional small-molecule drug discovery has historically depended on the occupancy-driven model, which necessitates the binding of a drug to a deep, hydrophobic pocket within an enzyme or receptor to sterically inhibit its function [6,7]. However, the upstream drivers of orthopaedic diseases often defy this structural paradigm. In osteoarthritis and inflammatory joint conditions, the pathology is orchestrated by transcription factors and non-enzymatic scaffolding proteins—such as NF-κB and HIF-2α—which drive the aberrant expression of catabolic enzymes like MMP-13 and ADAMTS-5, alongside a milieu of inflammatory cytokines [2,8]. Similarly, the uncoupling of bone remodeling in osteoporosis hinges on key lineage-determining factors like NFATc1, which dictates the osteoblast-osteoclast balance [9]. In the realm of oncology, bone sarcomas and metastatic lesions rely on tumorigenic hubs such as β-catenin and c-MYC to maintain stemness and remodel the osseous niche [10,11]. These proteins typically function as expansive signalling hubs lacking well-defined catalytic sites. Furthermore, they are often sequestered within super-enhancer-associated complexes or phase-separated biomolecular condensates, rendering them thermodynamically difficult for small molecules to inhibit and physically inaccessible to antibody-based therapies(Fig. 1c) [12]. The clinical consequence of this limitation is evident: while biologics targeting downstream effectors (e.g., TNF-α, IL-6, RANKL, or Sclerostin) have achieved success, the upstream pathological circuitry remains active [2,13]. This incomplete blockade often leads to therapeutic plateaus and the emergence of secondary resistance, as the disease network is merely attenuated rather than fundamentally rewritten.Moreover, the skeletal microenvironment is uniquely burdened by the accumulation of extracellular matrix debris and large intracellular protein aggregates, which pose a physical challenge often exceeding the processing capacity of the canonical proteasome [14,15].

The advent of Targeted Protein Degradation (TPD) has introduced a transformative pharmacological logic that promises to dismantle these historical barriers [[6], [7], [8]]. Departing from the conventional “occupancy-driven” model—which requires high-affinity, continuous binding to a functional site to inhibit activity—TPD operates on a catalytic, “event-driven” mechanism. By deploying bifunctional molecules, this strategy effectively converts a target protein's ligand into a degradation tag to harness cellular disposal machinery [[6], [7], [8]]. While the current landscape is dominated by Proteolysis-Targeting Chimeras(PROTACs) and molecular glues that recruit the ubiquitin-proteasome system (UPS), the field is rapidly expanding to encompass lysosome-dependent modalities(e.g., LYTACs, AUTACs) capable of clearing extracellular and aggregated targets [[16], [17], [18], [19]].These agents act as molecular bridges, inducing the formation of a ternary complex between the protein of interest and an E3 ubiquitin ligase. This proximity triggers the polyubiquitination of the target, marking it for rapid destruction via the endogenous 26S proteasome pathways. Crucially, because the degrader is not consumed in the process, a single molecule can iteratively destroy multiple copies of the pathogenic protein, offering a kinetic advantage that is particularly valuable in the orthopaedic context where drug delivery is limited. The efficacy of this approach has been robustly validated in oncology and immunology. Immunomodulatory drugs like lenalidomide and pomalidomide have demonstrated how molecular glues can reprogram the substrate specificity of the E3 ligase Cereblon (CRBN) to degrade “undruggable” zinc-finger transcription factors such as IKZF1 and IKZF3 [6]. Moreover, the successful degradation of scaffold proteins like the BET family or transcription factors like STAT3 confirms that “undruggability” is a limitation of the inhibitor modality, not the biological target itself [[6], [7], [8]].

Integrating TPD strategies into the orthopaedic landscape offers a unique opportunity to reclaim the vast array of molecular hubs previously deemed out of reach [5,7,8]. In the context of osteoarthritis and synovitis, PROTACs or molecular glues designed to degrade NF-κB or HIF-2α could simultaneously collapse multiple divergent inflammatory and catabolic cascades within chondrocytes and synovial fibroblasts, representing a significant leap beyond the linear inhibition of single cytokines. In osteoporosis and metabolic bone disorders, degraders targeting the master regulator NFATc1 could provide a precise, reversible modulation of osteoclast differentiation. Unlike current anti-resorptive therapies that risk “freezing” bone turnover by chronically locking the RANKL–RANK axis, TPD offers the potential to tune the upstream commitment of the osteoclast lineage. Furthermore, in osteosarcoma and bone metastases, TPD agents targeting c-MYC, β-catenin, or fusion oncoproteins could achieve a dual therapeutic effect: compromising tumor cell survival and stemness while simultaneously disrupting the tumor's ability to “educate” and exploit the bone marrow microenvironment. However, translating this promise into clinical reality requires navigating complex tissue barriers and identifying specific target-E3 pairs suitable for the skeletal environment. This is where the convergence of pharmacologically recalcitrant targets and complex tissue barriers defines the current frontier of orthopaedic medicine. By systematically dissecting the molecular nuances of these targets alongside the chemical biology of the TPD toolbox, and navigating the specific opportunities and risks within the skeletal context, this review establishes a comprehensive framework. We aim to bridge the gap between mechanistic understanding, deep learning-driven design, and multimodal data integration. Although our computational framework primarily draws from the mature datasets of PROTACs and molecular glues, we also delineate a forward-looking roadmap for integrating emerging lysosomal degradation strategies, to accelerate the translation of precision degradation therapies into clinical orthopaedic practice.

## 2 Targeted protein degradation in skeletal tissues: mechanisms, modalities, and bottlenecks

**Fig. 2:**
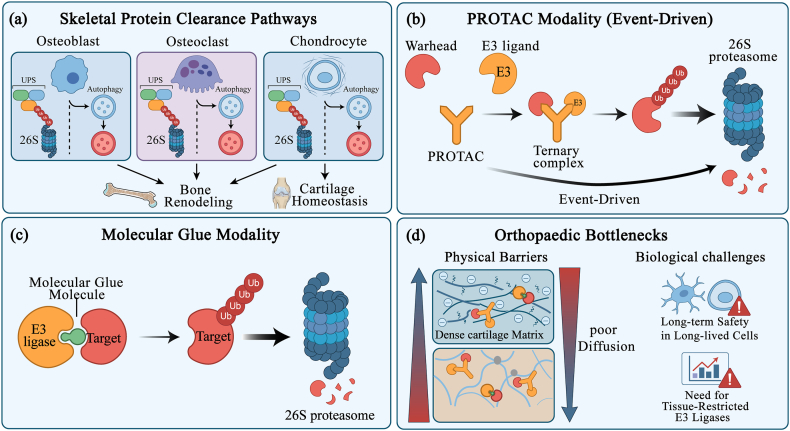
**Protein-clearance pathways and TPD modalities in skeletal tissues, with orthopaedic-specific bottlenecks.** (a)The ubiquitin–proteasome system (UPS) and autophagy–lysosome axis operate across key skeletal cell types (osteoblasts, osteoclasts, chondrocytes) to regulate bone remodeling and cartilage homeostasis, thereby shaping the degradable landscape in orthopaedic disease. (b) PROTACs are bifunctional molecules that bridge a target protein and an E3 ligase to form a ternary complex, promote ubiquitination, and trigger catalytic proteasomal degradation. (c) Molecular glues are monovalent small molecules that induce or stabilize neomorphic E3–target interfaces, enabling ubiquitination and degradation without a linker architecture. (d) Translation in orthopaedics is limited by in-tissue penetrability of beyond-Rule-of-5 degraders, dense extracellular matrices in cartilage and mineralized bone, scarcity of tissue-restricted E3 recruiters, and the amplified long-term safety burden imposed by slow skeletal turnover and long-lived resident cells. While not explicitly depicted, emerging lysosomal degraders (LYTACs/AUTACs) operate in parallel to these UPS-based mechanisms to clear extracellular and aggregated targets.

Targeted protein degradation (TPD) represents a paradigm shift in therapeutic interventions, leveraging endogenous protein degradation systems to catalyze the clearance of pathogenic proteins rather than merely inhibiting their activity [5,20]. This stands in stark contrast to traditional inhibitors, which require sustained, high-affinity occupancy of an active site to maintain pathway suppression [5,20]. In contrast, degradation agents function through an event-driven mechanism: once the target molecule is tagged and processed for degradation, the degradation agent can in principle dissociate and bind to other target molecules [5,20]. Within skeletal tissues, the intricate balance between the ubiquitin-proteasome system (UPS) and the lysosome/autophagy pathways is intimately linked to bone remodeling, cartilage homeostasis, and osteoimmunology, rendering them attractive yet delicate entry points for TPD [[21], [22], [23]]. Based on the microenvironmental barriers and undruggable targets illustrated in Fig. 1, this section elucidates the mechanistic basis for target deprotection (TPD) in bone and cartilage tissues. To fully appreciate its transformative potential in musculoskeletal medicine, it is essential to trace the logical progression of current therapeutic challenges. First, we outline the unique physicochemical and anatomical barriers inherent to the skeletal niche (Section 2.1). We then explore how the UPS (Section 2.2) and emerging non-proteasomal modalities—such as lysosome-targeting technologies for clearing extracellular aggregates (Section 2.3)—are harnessed to overcome these barriers. Finally, we contextualize these mechanisms within the delicate balance of bone homeostasis, delineating the specific safety and toxicity considerations required for orthopaedic applications (Section 2.4). Together, these subsections compare the characteristics of different TPD modalities and highlight how orthopaedic-specific opportunities and bottlenecks influence target selection, E3 ligase screening, and degrader design (Fig. 2).

### 2.1 Protein clearance pathways and TPD mechanisms in bone biology

The ubiquitin-proteasome system (UPS) and the lysosome/autophagy axis are master regulators of protein turnover in osteoblasts, osteoclasts, and chondrocytes, thereby critically shaping bone formation, resorption, and cartilage integrity(Fig. 2 a) [[21], [22], [23], [24]]. In osteoblast lineage cells, the tightly controlled ubiquitination and proteasomal degradation of transcription factors such as RUNX2, β-catenin, and SMADs provide a crucial mechanism for fine-tuning osteogenic differentiation and matrix production [21,22]. Conversely, in osteoclasts, a complex interplay of ubiquitin ligases and deubiquitinating enzymes regulates key mediators of osteoclastogenesis, cytoskeletal dynamics, and resorptive activity [24,25]. Meanwhile, in chondrocytes, these protein clearance mechanisms modulate adaptive responses to mechanical load, inflammatory cytokines, and cues for endochondral ossification [22,23]. Dysregulation of these finely tuned networks precipitates pathological phenotypes, including aberrant osteoclast activation, chondrocyte senescence, defective bone formation, and osteosarcoma progression [[21], [22], [23], [24]]. This underscores that protein turnover is not a mere housekeeping detail but a core, dynamic layer of skeletal biology, the manipulation of which holds immense therapeutic potential.

At a mechanistic level, the canonical UPS involves the sequential action of E1 (activating), E2 (conjugating), and E3 (ligating) enzymes, which collaborate to attach polyubiquitin chains onto target proteins, marking them for recognition and degradation by the 26S proteasome [20,21]. In parallel, selective forms of autophagy and lysosomal degradation—such as aggrephagy (for protein aggregates) and mitophagy (for damaged mitochondria)—handle larger complexes and organelles, while broader lysosomal pathways extend this clearance capacity to extracellular and membrane-associated proteins [18,19,23]. Emerging TPD modalities, exemplified by LYTACs and AUTACs, are specifically designed to co-opt these pathways by engaging autophagy receptors or components of the lysosome-associated machinery [18,19]. TPD agents exploit these pre-existing systems by forcibly juxtaposing a target protein with an E3 ligase or another degradation machinery component, thereby triggering ubiquitination or an alternative degradation tag and routing the target for destruction. Given that both the UPS and autophagy are intricately involved in the physiological remodeling of bone and cartilage, the design of TPD strategies must carefully consider how perturbing specific nodes within these systems will create ripple effects across cellular and tissue-level homeostasis.

### 2.2 PROTACs and molecular glues: Advantages and limitations of specific modalities

Proteolysis-Targeting Chimeras (PROTACs) offer a modular and versatile platform for redirecting E3 ubiquitin ligases toward previously intractable targets relevant to osteoarthritis, osteoporosis, and bone tumors. A typical PROTACs is a heterobifunctional molecule comprising three distinct elements: a ligand that binds the protein of interest (POI), a ligand that recruits a specific E3 ligase, and a chemical linker that spatially organizes the two binding moieties to facilitate the formation of a productive ternary complex [5,20]. This architecture allows medicinal chemists to repurpose validated warheads (e.g., inhibitors of kinases or nuclear receptors) and known E3 ligase binders (e.g., ligands for VHL, CRBN, or IAPs), while systematically exploring linker length, composition, and attachment points to optimize degradation efficiency and selectivity(Fig. 2 b) [5,20]. For orthopaedic indications, such PROTACs could, in principle, be designed to degrade inflammatory transcription factors, catabolic enzymes, or oncogenic drivers that lack traditional druggable catalytic pockets but for which small-molecule binders or fragment-derived ligands are available.

However, the relatively large molecular size, high polarity, and linker-dependent properties of many PROTACs raise legitimate concerns regarding their ability to penetrate dense bone matrix and avascular cartilage [3,26,27]. High molecular weight and polar surface area can impair oral bioavailability, tissue permeability, and intracellular access, particularly in contexts characterized by a thick extracellular matrix and limited convective transport, such as articular cartilage and the intervertebral disc. Furthermore, PROTACs activity is contingent upon the abundance, subcellular localization, and functional state of the recruited E3 ligase, which can vary significantly between osteoblasts, osteoclasts, chondrocytes, and bone marrow stromal cells [21,24]. Collectively, these factors necessitate that PROTACs design for orthopaedic applications must transcend conventional drug-likeness criteria (e.g., Lipinski's Rule of Five) to incorporate skeletal- and joint-specific considerations, including diffusion through cartilage, penetration into subchondral bone, and retention within the local microenvironment.

Molecular glue degraders represent a complementary TPD modality founded on distinct design principles [28,29]. Unlike PROTACs, which covalently link two ligands via a chemical linker, molecular glues are typically low-molecular-weight small molecules whose mechanism of action lies in inducing or stabilizing protein-protein interactions between E3 ligases and target proteins that are inherently weak or non-existent(Fig. 2c). By reshaping the molecular interfaces of ligases or targets, they create new complex surfaces that facilitate selective ubiquitination of otherwise undruggable substrates. The paradigm of clinically successful molecular glues—immunomodulatory imidazolidinyl dithiazoles (IMiDs) such as lenalidomide—have fundamentally transformed the treatment landscape for hematologic malignancies like multiple myeloma. These molecular glues induce the CRBN E3 ligase to recognize and degrade transcription factors (like IKZF1/3) that lack a classical small-molecule binding pocket, demonstrating highly selective degradation efficacy. Applying the molecular glue concept to orthopedics requires careful consideration of the expression profiles of bone- and cartilage-specific E3 ligases, target biological characteristics, and potential off-target degradation risks within the skeletal system. Additionally, advanced methods are needed to predict or rationally design inducible binding interfaces within relevant protein pairs.

For both PROTACs and molecular glues, the selection of the E3 ligase and its expression context are pivotal determinants of both efficacy and safety. Closely related E3 ligases can exhibit divergent expression patterns across skeletal cell types and developmental stages. Moreover, the behavior of an identical ligase-target pair may differ profoundly in osteoblasts, osteoclasts, or chondrocytes compared to its function in hematopoietic or epithelial cells. Consequently, a systematic and detailed understanding of the E3 ligase landscape within bone and cartilage—identifying which ligases can be harnessed without compromising essential skeletal functions—is a fundamental prerequisite for rational modality selection and degrader design [21,24].

### 2.3 Beyond the proteasome: Emerging TPD modalities for extracellular and aggregate targets

While PROTACs and molecular glues have revolutionized the targeting of intracellular substrates via the ubiquitin-proteasome system (UPS), a significant proportion of orthopaedic pathological drivers remain beyond the reach of the proteasome [16,30]. Specifically, the skeletal microenvironment is heavily influenced by extracellular matrix (ECM) proteins, membrane-bound receptors, and large insoluble aggregates, which physically exceed the narrow pore size of the 26S proteasome barrel [31]. To achieve holistic “microenvironmental reprogramming,” emerging modalities such as Lysosome-Targeting Chimeras (LYTACs) and Autophagy-Targeting Chimeras (AUTACs) represent critical complementary strategies to classical PROTACs [6,17].

LYTACs exploit cell-surface receptors (e.g., CI-M6PR or ASGPR) to trigger the internalization and lysosomal degradation of extracellular and membrane proteins [31,32]. In the context of osteoarthritis (OA) and rheumatoid arthritis, LYTACs hold the potential to degrade pro-inflammatory cytokine receptors or scavenge pathogenic ECM fragments from the synovial fluid—targets that are topologically inaccessible to cytosolic PROTACs [31]. In contrast, AUTACs and ATTECs (autophagy-tethering compounds) hijack the autophagy pathway through distinct mechanisms: AUTACs induce K63-linked polyubiquitin chains on target proteins, mimicking the autophagy labeling of damaged organelles, thereby being recognized by the autophagy receptor p62 and incorporated into autophagosomes [19]; ATTECs directly bind to both the LC3 protein and the target protein as dual ligands, anchoring substrates to the autophagosome membrane [33]. Both enable selective clearance of large intracellular substrates such as damaged organelles and protein aggregatesvia lysosomal degradation [31,33]. Given that autophagy dysfunction is a hallmark of chondrocyte senescence, these autophagy-based degraders offer a unique mechanism for clearing accumulated “cellular debris” in aged bone tissue [34].

Although these modalities are biologically promising, their integration with deep learning (DL) remains nascent compared to PROTACs. The lack of standardized datasets for lysosomal targeting motifs and the complexity of modeling protein-glycan interactions (crucial for LYTACs) currently limit the application of generative AI in this niche [[35], [36], [37]]. However, as DL models evolve to encompass broader chemical and biological spaces, extending the “AI-TPD” framework to include lysosomal and autophagic degradation will be the next logical frontier for orthopaedic precision medicine [[35], [36], [37]].

### 2.4 Orthopaedic-specific risks and design considerations

Beyond general pharmacokinetic and safety concerns, orthopaedic TPD faces unique bottlenecks imposed by the distinctive anatomy, biomechanics, and cell biology of skeletal tissues(Fig. 2 d). The limited vascularization of articular cartilage, the intervertebral disc, and certain regions of trabecular bone restricts the rate and extent of degrader delivery, increasing the likelihood of steep spatial gradients in target engagement [3,27,38,39]. The complex loading environment subjects cells and the extracellular matrix to cyclic mechanical stress, which can modulate cellular uptake, subcellular trafficking, and stress response pathways that crosstalk with the protein degradation machinery—for instance, by regulating ubiquitin-proteasome activity or autophagy flux via mechanosensitive adaptors such as HDAC6 and p62 [40,41]. Furthermore, bone and cartilage harbor long-lived, matrix-embedded cells, such as osteocytes and deep-zone chondrocytes, which can accumulate exposure to TPD drugs over years or decades—a long-term biological impact that is difficult to predict from short-term studies [21,24,25,42].

Adding another layer of complexity, the long-term or excessive degradation of key regulators in bone remodeling could inadvertently lead to skeletal fragility or impaired repair capacity. A target that is pathogenic in one compartment or disease context may perform essential homeostatic functions elsewhere.For example, while targeting the Wnt/β-catenin signaling axis is a compelling strategy for eradicating osteosarcoma stem cells, employing a ubiquitous E3 ligase (such as CRBN) to systemically degrade β-catenin poses a severe “on-target, off-tissue” risk. Such widespread degradation would simultaneously suppress osteoblastogenesis globally, potentially inducing catastrophic adynamic bone disease or rapid osteoporosis in adjacent healthy compartments [43]. Similarly, the E3 ligases recruited for TPD might have non-redundant, fundamental roles in osteoblast or osteoclast biology. In a disease like osteoporosis, where the therapeutic goal is the subtle rebalancing of bone formation and resorption over extended periods, even modest off-target or on-target effects in unintended cell types could translate into clinically relevant changes in fracture risk. Conversely, in aggressive bone tumors or metastases, a more profound degradation of oncogenic drivers may be justified, yet this must still be balanced against potential local and systemic skeletal toxicities.

These orthopaedic-specific risks imply that the critical decisions surrounding target selection, E3 ligase choice, modality (PROTACs vs. molecular glue), dosing regimen, and route of administration cannot be simply extrapolated from oncology or other fields. Instead, they must be explicitly optimized with reference to skeletal anatomy, microenvironmental barriers, and the dual objectives of therapeutic efficacy and the preservation of long-term structural integrity. In subsequent sections, we will explore how deep learning and multimodal data integration can help to prospectively predict these liabilities and guide the development of TPD strategies compatible with the unique demands of orthopaedic applications.

## 3 Deep learning and multimodal data for orthopaedic TPD

**Fig. 3:**
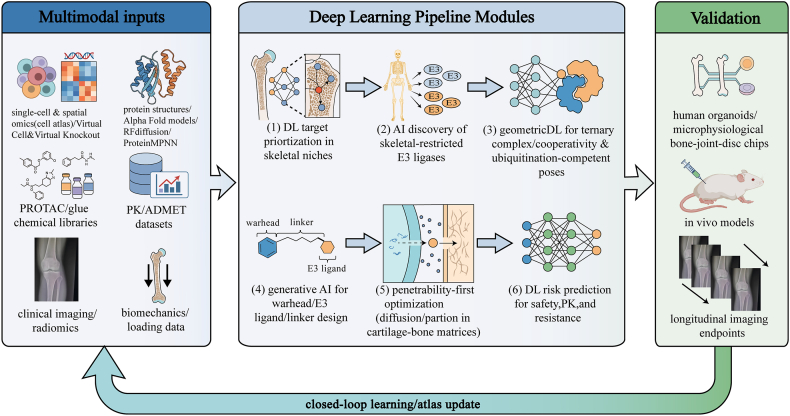
**Closed-loop deep learning pipeline for orthopaedic TPD discovery and optimization.** Schematic overview of a DL-enabled, multimodal workflow tailored to skeletal diseases. Multimodal inputs include single-cell and spatial omics atlases, structural models of targets/E3 ligases, degrader chemical libraries, PK/ADMET datasets, clinical imaging/radiomics, and biomechanics/loading information. These data feed sequential DL modules for (1) niche-conditioned target prioritization, (2) AI discovery of skeletal-restricted E3 ligases, (3) geometric scoring of ternary-complex cooperativity and ubiquitination-competent poses, (4) generative design of warheads, E3 binders, and linkers, (5) penetrability-first optimization within cartilage–bone matrices, and (6) prediction of safety, PK/PD, and resistance events. Experimental validation in microphysiological systems, organoids, and in vivo models, coupled with quantitative imaging endpoints, closes the loop by updating disease atlases and model parameters for iterative improvement.

Deep learning (DL) has moved from a supportive technology to a genuine rate-limiting enabler of targeted protein degradation (TPD) [44,45]. What distinguishes DL from classical step-wise cheminformatics is its ability to learn non-linear couplings across the pipeline—linking target context, ternary-complex geometry, degrader physicochemistry, and long-term risk in a single modeling space. In orthopaedics, this systems view is not a luxury. The skeletal niche combines “hard-to-reach” anatomy with “hard-to-drug” molecular hubs, and therefore demands computational strategies that simultaneously optimize what to degrade, how to recruit an E3, and whether a degrader can physically and safely operate inside bone-joint microenvironments. The following subsections first summarize what DL is already delivering for TPD in broader settings, then show how these modules must be re-parameterized for orthopaedic translation (Fig. 3).

### 3.1 AI-driven discovery: From multimodal mapping to target prioritization

While deep learning provides powerful optimization tools, the translational value of its outputs is inherently dependent on the biological context used for their interpretation. To design microenvironment-aware TPD strategies, it is paramount to integrate diverse data modalities that capture distinct facets of bone and joint pathobiology [46,47]. At the cellular level, single-cell and spatial transcriptomics have begun to delineate the complex ecosystems of subchondral bone, articular cartilage, the growth plate, and marrow niches. Deep learning analyses of these datasets unveil disease-enriched cell states and spatially restricted niches—such as inflammatory synovial fibroblast clusters at the cartilage-synovium interface or osteoclast-rich microenvironments within osteolytic lesions—that may harbor high-priority TPD targets [48].At the macroscopic level, clinical imaging and biomechanics add a critical phenotypic layer. Deep learning-based radiomics models extract quantitative features from X-ray, CT, and MRI scans, capturing subtle alterations in trabecular architecture, osteophyte morphology, or cartilage texture [49,50]. By fusing these imaging-derived phenotypes with molecular signatures, multimodal models aim to construct cross-scale maps that link molecular targets to clinically meaningful outcomes, such as slowing joint space narrowing or reducing vertebral fracture risk.To operationalize the integration of these diverse datasets, researchers are adopting a systematic, three-step computational workflow. First, spatial omics data are aligned to map the precise geographical distribution and expression gradients of specific E3 ligases and pathogenic targets across distinct histological zones (e.g., superficial versus deep articular cartilage). Second, matched high-resolution radiological features are extracted via micro-CT or MRI to quantitatively assess structural barriers, such as matrix porosity and bone mineral density. Third, deep learning-based late-fusion architectures integrate these cellular and radiological data layers. Representative studies applying such multimodal integration—for instance, combining spatial transcriptomics with MRI radiomics in osteoarthritis or osteosarcoma microenvironments [51,52]—demonstrate how these cross-scale maps successfully link molecular targets to clinically meaningful macroscopic outcomes, such as slowing joint space narrowing or reducing vertebral fracture risk. However, translating these correlative observations into actionable TPD targets requires systematic computational frameworks for functional prioritization and structural assessment—a challenge addressed by the following AI-driven strategies.

The foundational step in TPD involves identifying high-value targets—often transcription factors or scaffold proteins—that defy conventional druggability metrics. Deep learning frameworks integrate bulk and single-cell omics to pinpoint signaling hubs critical for pathological maintenance [46,47].Graph-based and attention-based models applied to regulatory networks can identify essential signaling nodes in osteoclasts or chondrocytes [53].However, merely identifying network hubs is insufficient to establish causality. Complementary *in silico* approaches—broadly termed ‘virtual knockout’ and ‘virtual cell modeling’—are emerging to advance this screening process from static topological analysis toward dynamic systems simulation [54,55].Network-based virtual knockout, which algorithmically removes a node from disease-associated gene regulatory or protein-protein interaction networks, can help prioritize targets whose deletion is predicted to destabilize pathological modules while sparing core physiological networks [56].More recently, virtual cell models—constructed by integrating single-cell multi-omics data with foundation models pre-trained on large-scale transcriptomic atlases (e.g., scGPT, Geneformer)—have shown preliminary promise in simulating gene expression perturbations following target perturbation [57]. However, their capacity to quantitatively predict the full phenotypic consequences of specific protein degradation—particularly in complex orthopaedic cell types such as chondrocytes or osteoclasts—remains highly exploratory and necessitates extensive experimental validation.Nevertheless, when integrated with structure-informed substrate assessment (e.g., degron prediction, surface accessibility), these emerging computational strategies offer a conceptual framework for filtering false-positive targets that, while centrally networked, may possess redundant or compensatory functions. Distinct from functional validation, protein language models and structure-informed classifiers concurrently assess structural viability, effectively distinguishing disordered, disease-associated bystanders from structurally viable TPD substrates [[57], [58], [59], [60]].

Once targets are prioritized, selecting an appropriate E3 ubiquitin ligase is critical. The reliance on ubiquitous ligases (e.g., CRBN, VHL) in current TPD strategies poses a risk of systemic toxicity in chronic orthopaedic conditions [44,61].To mitigate this, the field is pivoting toward AI-driven discovery of ‘skeletal lineage-enriched’ E3s [46,47,61]. To achieve this, graph neural networks (GNNs) are employed to navigate complex protein-protein interaction (PPI) and gene co-expression networks. By analyzing the topological features of these networks alongside single-cell atlases (the underlying principle), GNNs can systematically pinpoint tissue-restricted E3 ligases—such as specific TRIM or FBXO family members—that are exclusively active within the joint microenvironment (the practical outcome). Subsequent AlphaFold-based structural modeling screens these candidates for “ligandable” pockets, transforming serendipity into a systematic, data-driven screening process [62].

### 3.2 Generative design and rational engineering of degraders

Downstream of target and ligase selection, deep learning is redefining the molecular engineering of degraders, shifting the paradigm from empirical screening to computation-guided “rational design.”

The linker moiety, historically viewed as a passive connector, is a critical determinant of physicochemical properties and tissue permeability in orthopaedics. Traditional trial-and-error optimization is labor-intensive and often yields compounds with poor diffusivity in dense cartilage matrices. Deep learning treats linker design as a multi-objective optimization problem. Geometric Deep Learning (GDL) models predict the optimal linker length and rigidity required to minimize the free energy of the Target-Linker-E3 ternary complex [63]. Furthermore, generative models such as graph-based variational autoencoders (VAEs) and diffusion models have been adapted to generate PROTACs linkers with specific physicochemical constraints—such as reduced topological polar surface area (TPSA)—to enhance passive diffusion through avascular chondral matrices, exemplified by recent equivariant graph transformer diffusion approaches for PROTACs design [64].

A major hurdle in applying these architectures to orthopaedics is the “small data” challenge compared to oncology [65,66].To bridge this gap, Transfer Learning allows foundational models pre-trained on vast general-purpose databases (e.g., AlphaFold DB, ChEMBL) to be fine-tuned on limited, high-quality orthopaedic datasets. For instance, geometric deep learning models can learn the rules of ternary complex stability from oncology data and transfer these representations to predict efficacy in the chondrocyte proteome [45,67].Meta-learning algorithms further empower ligand prediction for skeletal lineage-enriched E3s without requiring prohibitive amounts of *de novo* data.

For ‘undruggable’ targets that lack classical binding pockets—such as HIF-2α or specific interfaces of the NF-κB complex—the paradigm of *de novo* protein design, pioneered by the David Baker group, offers a revolutionary solution. While traditional virtual screening relies on existing chemical libraries, Baker's deep learning architectures, such as RFdiffusion (a generative model based on diffusion processes) and ProteinMPNN, enable the hallucination of entirely new protein binders with atomic-level precision [68,69]. For structurally arduous targets like HIF-2α or specific interfaces of the NF-κB complex, which lack deep hydrophobic pockets, RFdiffusion can be steered to design novel mini-protein binders or macrocyclic peptides that conform perfectly to these flat surfaces. Furthermore, Baker's team has demonstrated the capability to computationally design rigid scaffolds that position functional motifs with sub-angstrom accuracy. In the context of PROTACs, this technology offers a potential future pathway for generating high-affinity, rigid protein-based E3 ligase or target-binding moieties, which is particularly valuable for targets lacking small-molecule ligands. It is noteworthy, however, that current *de novo* protein design methods have not yet been applied to designing small-molecule linkers or drug-like E3 ligase recruiters; their implementation in PROTACs engineering remains at the conceptual stage. Nevertheless, the paradigm shift from “finding” to “creating” ligands exemplified by Baker's work points the way forward for deep learning-driven orthopedic TPD development, even if its direct application remains some time away.For molecular glues, deep learning analysis of chemogenomic profiles aids in identifying compounds that induce novel E3-target interactions [70]. Collectively, these computational pipelines are steering orthopaedic TPD away from high-throughput trial-and-error toward a focused, hypothesis-driven paradigm.

It is worth noting that current deep learning architectures are predominantly optimized for UPS-based PROTACs due to the availability of ternary complex structural data [35,71]. However, AI-driven design for emerging LYTACs and AUTACs remains a frontier challenge. Designing LYTACs requires predicting complex protein-glycan interactions—often overlooked by standard AlphaFold pipelines—while AUTACs optimization demands modeling phase separation dynamics and autophagosome recruitment. Future algorithms must therefore evolve from predicting rigid docking events to simulating the fluid assembly and membrane-recognition processes essential for these lysosomal modalities.

### 3.3 Deep learning-driven design of bone and cartilage targeted delivery systems

**Fig. 4:**
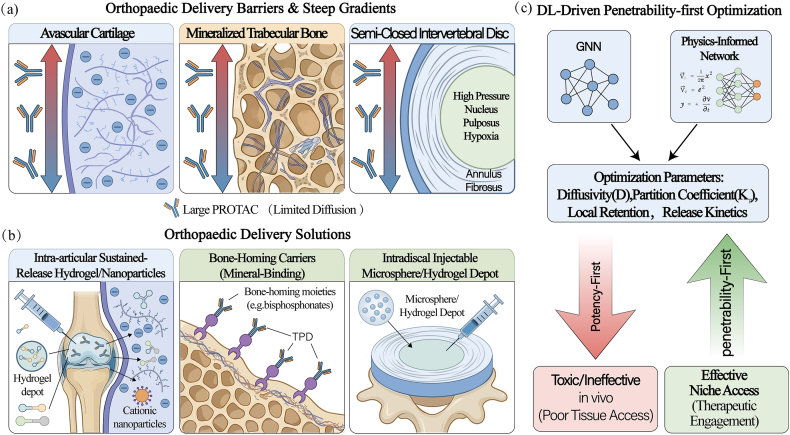
**Deep Learning-Driven Design of Bone and Cartilage Targeted Delivery Systems.** (a)Distinct physical and biochemical barriers across avascular cartilage, mineralized trabecular bone, and the semi-closed intervertebral disc create steep spatial gradients and severely limit diffusion of large, flexible degraders. (b) Orthopaedic delivery strategies that convert systemic limitations into niche access, including intra-articular hydrogels/nanoparticles, bone-homing carriers that exploit mineral affinity, and intradiscal injectable depots for sustained local exposure. (c) DL-assisted optimization of diffusivity, partitioning, retention, and release kinetics reorders discovery priorities from potency-first to penetrability-first, aligning ternary-complex productivity with feasible in-tissue pharmacology for durable disease modification.

Although Targeted Protein Degradation (TPD) technologies demonstrate exceptional efficacy *in vitro*, their clinical translation in orthopaedics is fundamentally impeded by a “physicochemical mismatch”: degraders such as PROTACs typically possess high molecular weights and pronounced polarity, resulting in poor passive permeability and challenging pharmacokinetics [16,72]. As illustrated in Fig. 4a, these characteristics render passive diffusion through the dense, negatively charged matrix of avascular cartilage or the mineralized lattice of trabecular bone nearly impossible [73]. Consequently, the field must undergo a paradigm shift from traditional “potency-first” screening toward a “penetrability-first” strategy (Fig. 4c). This shift necessitates the precise, deep learning-enabled engineering of delivery vehicles that can convert systemic limitations into niche-specific access [16].

To address the barriers in mineralized bone tissue, the core challenge is achieving high-affinity binding to hydroxyapatite (HA) while enabling stimuli-responsive release. Deep learning models, particularly Graph Neural Networks (GNNs), are replacing trial-and-error synthesis to rationalize this process. By integrating Molecular Dynamics (MD) simulations, GNNs can virtually screen functional groups—such as the bisphosphonate moieties shown in Fig. 4b—to predict their binding free energy with HA surfaces [37,74]. Advanced models further incorporate pH-sensitivity parameters, simulating protonation state changes in the acidic osteoclast lacunae to ensure cargo release occurs exclusively in pathologically active remodeling regions [75,76].

For soft tissues like articular cartilage and intervertebral discs, drug delivery faces a dual “spatial–charge” barrier imposed by dense proteoglycan networks (Fig. 4a, left and right panels) [77]. Here, Physics-Informed Neural Networks (PINNs) offer a distinct advantage over “black-box” models. As depicted in Fig. 4c, PINNs embed fundamental physical laws—such as the Nernst–Planck equation and Donnan equilibrium theory—directly into the loss function [78]. This allows for the precise simulation of diffusion–reaction kinetics (*D*) and partition coefficients (K_p_) of cationic nanocarriers within negatively charged nanoporous matrices.Ultimately, this computational framework enables co-evolutionary design: the simultaneous optimization of a degrader's physicochemical properties and its carrier's transport mechanics. By shifting the optimization goal from pure potency to “effective niche access” (Fig. 4c), these AI-driven strategies ensure that supramolecular TPD systems can penetrate the matrix-isolated core of lesions, a prerequisite for true microenvironmental reprogramming [5].

### 3.4 Modeling safety, pharmacokinetics, and resistance events in the skeletal context

**Fig. 5:**
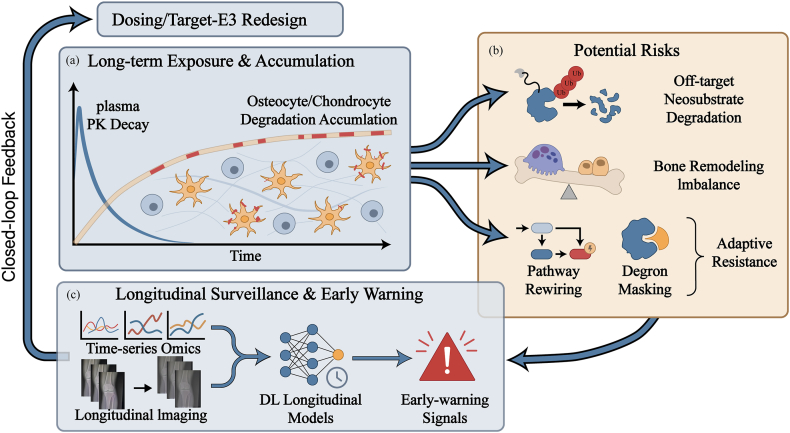
Closed-loop AI surveillance framework for modeling safety, pharmacokinetics, and resistance in orthopaedic TPD. (a) Kinetic Decoupling: The schematic graph illustrates the critical mismatch between the rapid decay of plasma pharmacokinetics (PK, blue line) and the sustained accumulation of protein degradation effects (orange dotted line) within long-lived skeletal cells (osteocytes/chondrocytes). This “memory effect” necessitates long-term modeling beyond systemic clearance. (b) Potential Risks: Prolonged tissue exposure amplifies specific orthopaedic liabilities, including off-target neosubstrate degradation and bone remodeling imbalance (disruption of osteoblast/osteoclast equilibrium). Additionally, adaptive resistance mechanisms are highlighted, specifically pathway rewiring and degron masking, which allow cells to evade degradation. (c) Longitudinal Surveillance & Feedback: A deep learning-enabled module integrates time-series omics and longitudinal imaging (e.g., X-ray/MRI sequences) to detect early-warning signals. These insights form a closed loop (left arrow), feeding back into the pipeline to guide the redesign of dosing strategies or Target-E3 pairs, thereby preempting clinical failure.

The event-driven pharmacology inherent to TPD raises unique concerns regarding off-target degradation, long-term biological consequences, and adaptive resistance, particularly within long-lived skeletal tissues [79,80]. Unlike traditional occupancy-based inhibitors, degraders often exhibit a profound kinetic decoupling between systemic clearance and tissue-level effects. As vividly illustrated in the pharmacokinetic profile in Fig. 5a, while the degrader's plasma concentration follows a rapid decay curve (blue line), its catalytic mechanism leads to a sustained accumulation of degradation events (orange curve) within resident osteocytes and deep-zone chondrocytes. This “memory effect” implies that functional repercussions may persist long after the drug has been cleared from systemic circulation, complicating the relationship between dose, exposure, and clinical effect.

Deep learning models trained on pharmacogenomic profiles, toxicity datasets, and longitudinal omics measurements can assist in predicting these specific liabilities *in silico*.Specifically, advanced deep learning algorithms can leverage skeletal single-cell RNA sequencing (scRNA-seq) atlases to map the precise expression networks of proposed target-E3 pairs [81,82]. By identifying how these proteins co-exist across non-diseased skeletal lineages, the AI acts as a “digital safety filter.” It can be trained to automatically downgrade or reject degrader designs that hijack E3 ligases heavily relied upon by essential homeostatic cells (e.g., healthy osteoblasts), thereby proactively screening out candidates with high off-tissue toxicity risks before experimental testing [83]. Fig. 5b delineates the primary risks that such models must anticipate: (1) Off-target neosubstrate degradation, where prolonged intracellular residence increases the probability of recruiting non-cognate proteins; and (2) Bone remodeling imbalance, visualized as the disruption of the delicate equilibrium between osteoblastic formation and osteoclastic resorption. Pharmacokinetic and pharmacodynamic (PK/PD) models augmented by deep learning can simulate these scenarios by incorporating variables such as local blood flow, matrix density, and cell-specific uptake rates, thereby predicting spatially resolved target occupancy and potential adverse outcomes on bone microarchitecture [80,84].

Adaptive resistance presents another significant challenge, especially for malignant or highly plastic cell populations residing within bone niches [85]. Beyond identifying generic mutation acquisition, deep learning models analyzing longitudinal multi-omics data are now being designed to map the evolutionary trajectories of specific resistance mechanisms depicted in Fig. 5b. By utilizing time-series AI architectures (such as recurrent neural networks), algorithms can detect early-stage cellular adaptation long before it manifests as clinical failure [86].Crucially, these predictive features must be contextualized within specific orthopaedic pathobiology. For instance, when designing degraders to correct remodeling abnormalities in osteoporosis, deep learning models can be trained to track characteristic pathway rewiring, such as detecting the compensatory secondary upregulation of Wnt/β-catenin or BMP signaling networks following prolonged osteoclast-targeted degradation [87]. Similarly, in the context of bone tumors like osteosarcoma, models can identify specific transcriptomic signatures indicative of therapeutic evasion. Beyond the rapid amplification of multidrug resistance (MDR) efflux pumps [88], DL algorithms can predict precise genomic point mutations leading to degron masking (where structural modifications obscure the ubiquitin ligase recognition motif), or the epigenetic silencing of the recruited E3 ligase itself—a frequent and critical mode of PROTAC resistance [89].Ultimately, identifying these precise mechanisms *in silico* is critical for guiding the development of degrader series that are resilient to evolutionary pressures. Rather than passively waiting for relapse, this AI-driven foresight enables the proactive design of “resistance-proof” clinical strategies, such as rationally alternating E3 ligase recruiters (e.g., dynamically switching from CRBN to VHL ligands) or deploying multi-targeted degraders to preempt skeletal therapeutic escape [90].

Building on this, the objective of these computational endeavors is to move from passive observation to active intervention, establishing a closed-loop surveillance framework (Fig. 5c). By integrating time-series omics and longitudinal clinical imaging, DL algorithms can generate early-warning signals of toxicity or resistance [44]. Crucially, as emphasized by the feedback loop in Fig. 5, these signals serve as actionable inputs to inform the rational redesign of dosing regimens or Target-E3 pairs. This proactive approach enables safety and resistance issues to be addressed through design modifications long before commitments are made to large-scale clinical testing, ensuring the long-term structural integrity of the skeletal system.

## 4 Disease applications and translational roadmap

**Fig. 6:**
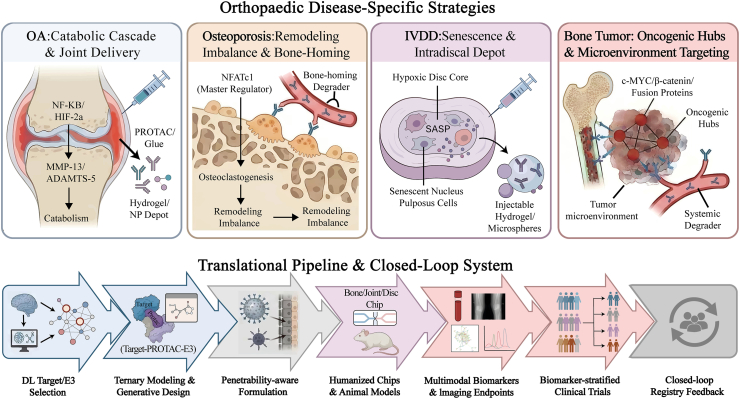
Orthopaedic indication-specific TPD strategies and a translational roadmap.

Orthopaedic diseases exhibit profound etiological heterogeneity, where distinct combinations of inflammatory, metabolic, mechanical stress, and oncogenic drivers collectively dictate the applicability and potential risks of Targeted Protein Degradation (TPD) strategies [91]. Specifically, osteoarthritis is primarily driven by low-grade inflammation and matrix catabolism; osteoporosis stems from the uncoupling of bone formation and resorption; intervertebral disc degeneration is dominated by a harsh, ischemic microenvironment; and bone tumors and their metastases rely on specific oncogenic programs and the bone ecological niche [[92], [93], [94], [95]]. Given this diversity, developing a universal TPD solution is not feasible. Instead, it must be replaced by personalized disease paradigms that enable the precise alignment of molecular targets, E3 ligases, delivery tools, and clinical endpoints with specific indications(Fig. 6).We have constructed a systematic target-evidence chain based on known targets and E3 ligase selectivity for different orthopedic diseases, combined with AI-analyzed available data types, as shown in Table 1.

**Table 1:** Multimodal data resources for AI-Driven target discovery in orthopaedic microenvironments.

Indication	Potential Therapeutic Target (POI)	Preferred E3 Ligase (Source/Rationale)	Key Data Modalities for AI Model Training
Osteoarthritis (OA)	HIF-2α (Catabolic regulator)	VHL (Hypoxia-induced expression)	scRNA-seq (Chondrocytes), Spatial Transcriptomics, Histopathology Images
Osteoporosis (OP)	BTK/HPK1 (Osteoclastogenic signaling)	CRBN/VHL (Lineage-enriched expression)	Proteomics (Osteoclasts), scRNA-seq, PPI Networks, Crystal Structures
Intervertebral Disc Degeneration (IVDD)	NLRP3 (Inflammasome component)	VHL (Hypoxic nucleus pulposus niche)	Phospho-proteomics, scRNA-seq, Metabolomics Data
Bone Tumors	HIF-1α/c-MYC (Oncogenic drivers)	VHL/CRBN (Tumor microenvironment)	TCGA Genomics, IHC Images, Microarray Data

To rationally navigate this diversity and guide the preclinical examples discussed below, selecting the most appropriate Target-E3 ligase combination for any orthopaedic condition must adhere to four core criteria: (1) Pathological Essentiality and Specificity: The target must be a non-redundant driver of the disease state with minimal homeostatic roles in healthy skeletal niches. (2) Tissue-Restricted E3 Expression: To prevent systemic toxicity, the recruited E3 ligase should ideally exhibit skeletal lineage-enriched expression (e.g., utilizing bone-specific E3s rather than ubiquitous CRBN/VHL when treating chronic, non-malignant conditions like OA or osteoporosis). (3) Subcellular Co-localization: The target and E3 ligase must robustly co-exist in the same subcellular compartment within the diseased cell. (4) Structural Ligandability: Both proteins must possess pockets or surfaces amenable to binder development, facilitating a thermodynamically stable ternary complex.

Top tier: representative TPD opportunities across major skeletal diseases. Osteoarthritis features inflammatory–catabolic cascades driving cartilage erosion, favoring intra-articular sustained-release degrader depots. Osteoporosis is dominated by remodeling imbalance and osteoclast/osteoblast signaling hubs, motivating bone-homing and remodeling-targeted degraders. Intervertebral disc degeneration centers on hypoxic, senescent disc niches with restricted access, highlighting intradiscal injectable depots. Bone tumors/metastases depend on oncogenic transcriptional and epigenetic hubs within the marrow niche, supporting systemic degraders with microenvironmental targeting. Bottom tier: an end-to-end translation pathway from DL-guided target/E3 nomination and ternary engineering, through penetrability-aware chemistry and delivery, to preclinical skeletal models, multimodal biomarkers and imaging endpoints, biomarker-stratified early-phase trials, and registry-based closed-loop refinement.

### 4.1 Osteoarthritis: Dual targeting of inflammation and matrix degradation

Osteoarthritis (OA) is characterized by chronic low-grade inflammation, upregulated expression of catabolic enzymes, and aberrant interactions between articular cartilage, subchondral bone, and the synovium [92]. Under the combined influence of mechanical load and pro-inflammatory cytokines such as IL-1β and TNF, transcriptional pathways like NF-κB and AP-1 are activated within chondrocytes and synovial fibroblasts [92]. This subsequently upregulates the expression of proteolytic enzymes, including matrix metalloproteinases (MMPs) and aggrecanases, leading to the progressive destruction of the cartilage matrix. Concurrently, subchondral bone sclerosis and osteophyte formation further disrupt joint biomechanics, creating a self-perpetuating cycle of damage [96].

TPD offers a novel strategy to simultaneously intervene against both inflammatory transcription factors and matrix-degrading enzymes [20]. At the upstream level, degrading key inflammatory transcription factors or signal transduction proteins—such as components of the NF-κB pathway, AP-1 subunits, or their upstream kinases—can suppress the transcriptional activity of catabolic mediators in cartilage and synovium. At the downstream level, utilizing PROTACs or molecular glues to degrade specific MMPs or ADAMTS family members could provide a more durable blockade of the matrix degradation process compared to traditional occupancy-based inhibitors, particularly if cell-type or region-specific degradation can be achieved. For instance, a recently developed BTK-targeting degrader by Huang et al. (**Target: Bruton's tyrosine kinase [BTK]; E3 ligase: CRBN; Degrader type: small-molecule PROTAC**) has progressed to preclinical evaluation. Key preclinical findings demonstrated that it effectively degraded BTK *in vitro*, potently inhibited RANKL-induced osteoclastogenesis, and significantly alleviated inflammatory bone destruction in murine models [97,98]. Additionally, preclinical senolytic PROTACs such as PZ15227 (**Target: BCL-xL; E3 ligase: CRBN; Degrader type: small-molecule PROTAC**) have shown promise in selectively clearing senescent cells, offering a blueprint for rejuvenating the aged osteoarthritic joint microenvironment [99].Leveraging deep learning-driven target prioritization holds promise for identifying the most effective combinations of upstream and downstream targets to suppress catabolic signaling within an acceptable therapeutic window.Beyond intracellular targets, the application of LYTACs offers a theoretical advantage in OA by directly scavenging extracellular pro-inflammatory cytokines or their membrane-bound receptors from the synovial fluid, thereby intercepting catabolic signals before they transduce intracellularly.The delivery of TPD therapeutics to the OA joint presents both challenges and opportunities. The avascular nature of articular cartilage limits access for systemically administered drugs, but it also makes local intra-articular injection a highly attractive route of administration [100]. Coupling degraders with hydrogel depots, nanoparticles, or other sustained-release systems could maintain effective drug concentrations within the cartilage and synovium while minimizing systemic exposure. Computational pharmacokinetic models based on joint anatomy, synovial fluid dynamics, and cartilage permeability can inform rational dosing regimens and formulation design. For clinical translation, multimodal biomarkers—including MRI-based measures of cartilage thickness and texture, quantitative subchondral bone changes, and soluble markers of matrix metabolism—will be crucial for assessing whether the reprogramming effects of TPD on the joint microenvironment translate into genuine structural improvement.

### 4.2 Osteoporosis: Rebalancing bone formation and resorption

The essence of osteoporosis is an imbalance in bone remodeling, a loss of homeostasis between osteoblast-mediated bone formation and osteoclast-driven bone resorption, a process co-regulated by endocrine, immune, and mechanical signals [93]. Factors such as aging, sex hormone deficiency, and glucocorticoid excess can lead to enhanced osteoclast activity and diminished osteoblast function, resulting in trabecular thinning, increased cortical porosity, and a significantly elevated fracture risk. Current therapeutic strategies are largely dichotomous: inhibiting bone resorption (e.g., bisphosphonates, denosumab) or promoting bone formation (e.g., teriparatide, romosozumab) [93]. However, long-term use is often accompanied by safety concerns, and complete restoration of bone quality remains elusive.

TPD technology offers a new avenue for resetting bone metabolic balance. On the osteoclast front, developing degraders targeting key regulators of their differentiation and function, such as NFATc1, c-Fos, or TRAF6, could enable more precise inhibition of osteoclastogenesis [91]. This approach may avoid the compromised immune surveillance associated with broadly blocking upstream signals like RANKL. On the osteoblast front, degrading negative regulators of the Wnt signaling pathway, such as sclerostin or DKK1, could release the brake on bone formation and promote osteoblastic activity. For example, CL144 (**Target: MDM2; E3 ligase: CRBN; Degrader type: small-molecule PROTAC**) has shown significant translational potential for promoting bone formation. Key preclinical findings revealed that CL144 stabilizes p53 in osteoblasts, thereby promoting their differentiation and matrix synthesis, which led to significantly improved bone mass and accelerated healing outcomes in ovariectomized and bone defect mouse models [101].This finding suggests that an event-driven protein degradation strategy may hold greater potential than traditional receptor agonists. This finding suggests that an event-driven protein degradation strategy to promote bone anabolism may hold greater potential than traditional receptor agonists. Deep learning models that integrate single-cell data from osteoblast and osteoclast lineages, structural features, and degradability information are poised to identify optimal target-E3 pairs that maximize bone gain while minimizing off-target risks.

Given that osteoporosis is a systemic, chronic condition, the pharmacokinetic properties, skeletal distribution, and long-term safety of therapeutic degraders are particularly critical. Systemically administered degraders will encounter a complex bone microenvironment, encompassing highly remodeled trabecular bone and relatively quiescent cortical bone compartments. AI-enhanced PK/PD models that incorporate bone turnover rates, mineral binding affinity, and regional blood flow can simulate cumulative drug exposure in different bone compartments, informing dose adjustment [91]. For efficacy assessment, imaging-based bone mineral density, microstructural indices (e.g., from DXA, HR-pQCT), and vertebral fracture risk assessment are natural clinical endpoints for determining whether TPD improves bone strength. Given the risks associated with excessive suppression of resorption or over-stimulation of formation—such as adynamic bone disease—early-phase clinical trials will require carefully escalated dosing strategies and close monitoring of the dynamic changes in bone formation and resorption markers.

### 4.3 Intervertebral disc degeneration: Intervening in an ischemic microenvironment

Intervertebral disc degeneration (IVDD) is a pathological process driven by mechanical overload, limited nutrient supply, chronic hypoxia, and inflammatory responses within a confined, ischemic microenvironment [94]. Nucleus pulposus and annulus fibrosus cells persist under harsh conditions of acidic pH, high osmolarity, and accumulated metabolic waste, leading to matrix breakdown, cellular senescence, and cell death. This very microenvironment constitutes a major barrier to drug penetration and sustained target engagement, explaining the limited success of most pharmacological interventions in halting IVDD progression [94].

Applying TPD to IVDD treatment requires first overcoming the dual challenges of delivery and the hostile microenvironment. Biologically, potential targets include key transcriptional regulators and signaling proteins that drive catabolic and senescent programs in disc cells, such as NF-κB pathway components, crucial MAPK kinases, or senescence-associated pathway molecules. These targets are often recalcitrant to effective modulation by traditional inhibitors. Theoretically, degrading these nodal proteins could reduce the production of degradative enzymes and inflammatory mediators, while also mitigating the senescence-associated secretory phenotype (SASP).Looking further ahead, autophagy-based degraders (AUTACs)—which have been designed to clear protein aggregates and damaged organelles in other cellular contexts—could conceivably be repurposed to eliminate the oxidized proteins and lipofuscin that accumulate in senescent disc cells and resist proteasomal degradation [19,33]. However, this application remains entirely conceptual and has not yet been experimentally explored in the IVDD field. As outlined in Sections 3.1, 3.2, deep learning analysis of disc-specific single-cell and spatial omics data could, in principle, help identify the cell states and signaling circuits most amenable to TPD intervention, while structural modeling could inform the design of degraders compatible with the aberrant proteostasis of the degenerating disc.

Senolytic TPD strategies have shown promise in other disease settings. Senolytic TPD strategies have shown preclinical promise in addressing this. For example, PZ15227 (**Target: BCL-xL; E3 ligase: CRBN; Degrader type: small-molecule PROTAC**) was developed to selectively clear senescent cells [99]. Subsequently, 753b (**Target: dual BCL-xL/BCL-2; E3 ligase: VHL; Degrader type: small-molecule PROTAC**) was reported with potent senolytic activity [102]. Key preclinical findings from a recent orthopaedic conference (ORS 2024) highlighted that a senolytic PROTAC based on the 753b scaffold effectively cleared senescent nucleus pulposus cells and significantly alleviated disc degeneration in an aged mouse model, providing direct preclinical evidence for senolytic TPD strategies in IVDD therapy.

Regarding delivery strategy, employing advanced carrier systems is paramount. Encapsulating degraders within hydrogels, microspheres, or injectable biomaterial scaffolds for intradiscal injection can provide sustained local drug exposure within the nucleus pulposus while reducing systemic leakage and exposure [103]. However, the harsh physicochemical conditions inside the disc place higher demands on the stability of the degrader, its release kinetics, and the safety of the carrier material. Computational models that integrate the disc's mechanical properties, fluid transport, and degrader diffusion and metabolism can aid in designing delivery formulations that achieve effective target engagement without exacerbating structural damage. For efficacy assessment, a combination of imaging endpoints—such as disc height and MRI-based signal intensity and composition—alongside biomechanical testing and pain-related outcome measures, will be necessary to collectively evaluate whether TPD can effectively reverse the pathological trajectory of disc degeneration.

### 4.4 Bone tumors and bone metastases

Primary bone tumors (e.g., osteosarcoma, Ewing's sarcoma, chondrosarcoma) and bone metastases from cancers such as breast, prostate, and lung cancer typically exhibit complex oncogenic signaling networks and therapy-induced resistance mechanisms, against which current treatments often provide only partial and transient efficacy [104]. In these diseases, the bone microenvironment provides a rich source of growth factors, cytokines, and extracellular matrix signals that collectively support tumor cell survival and therapeutic evasion [105]. Although oncogenic transcription factors, fusion proteins, epigenetic regulators, and anti-apoptotic proteins are key molecular drivers of these processes, many lack well-defined pockets for traditional small-molecule inhibitors or can rapidly develop resistance through mutation and pathway rewiring.

TPD offers a novel approach to directly degrade these oncogenic and resistance drivers within the bone metastatic niche. PROTACs targeting fusion oncoproteins, aberrant transcription factors, or epigenetic modifiers has demonstrated potential in various non-orthopaedic malignancies, and analogous strategies are equally applicable to primary bone tumors and metastases. Furthermore, molecular glue that reprograms E3 ligases to target traditionally undruggable oncoproteins may offer a more elegant degradation strategy, particularly useful where PROTACs is hampered by its molecular size or delivery limitations [20]. As detailed in Sections 3.1, 3.2, deep learning-assisted structural modeling helps identify targetable protein interfaces and predict the spatial configuration of ternary complexes, while multi-omics and single-cell data derived from both tumor and stromal compartments can systematically reveal tumor vulnerabilities and potential resistance escape routes. Immunomodulatory drugs like lenalidomide, which function as molecular glues altering the substrate spectrum of the CRBN E3 ligase to induce degradation of key transcription factors IKZF1/3 in B-cells and plasma cells, represent the first clinical success stories of “reprogramming the ubiquitination targeting spectrum.” Their efficacy in multiple myeloma, which involves profound reduction of tumor burden coupled with improvement in the bone marrow microenvironment and osteolytic lesions, provides the most mature paradigm for the clinical translation of TPD within a bone microenvironment. This success also sets a critical precedent for TPD strategies targeting oncogenic transcription factors or fusion proteins in bone tumors/metastases. Clinical-stage androgen receptor (AR) and estrogen receptor (ER) degraders further demonstrate that systemically administered TPD can achieve meaningful tumor control in heavily pre-treated metastatic castration-resistant prostate cancer (mCRPC) and ER-positive breast cancer—both diseases where bone is a predominant site of metastasis—while maintaining an acceptable safety profile. For instance, bavdegalutamide/ARV-110 (**Target: Androgen Receptor; E3 ligase: CRBN; Degrader type: small-molecule PROTAC**) and vepdegestrant/ARV-471 (**Target: Estrogen Receptor; E3 ligase: CRBN; Degrader type: small-molecule PROTAC**) have transitioned from preclinical success to advanced clinical trials. Key preclinical and clinical findings demonstrated that these systemically administered degraders achieve meaningful tumor control, induce profound target knockdown, and elicit radiographic responses in heavily pre-treated metastatic castration-resistant prostate cancer and ER-positive breast cancer—both of which are conditions heavily reliant on the bone metastatic niche [106,107]. Additionally, the dual-specific BCL-xL/BCL-2 degrader 753b further validates the potential of TPD in overcoming anti-apoptotic resistance networks commonly encountered in malignant bone diseases [102].

Given the aggressive nature of malignant bone diseases, TPD is unlikely to be used as monotherapy but will need to be integrated into multimodal treatment plans encompassing surgery, radiotherapy, chemotherapy, targeted therapy, and immunotherapy. In this context, degraders could be deployed to eliminate key resistance nodes, thereby re-sensitizing tumors to existing therapies, or to disrupt tumor-bone microenvironment crosstalk that drives osteolysis and skeletal-related events. However, the therapeutic window between effective tumor control and unacceptable skeletal toxicity—particularly in weight-bearing bones—may be narrow. Consequently, the design of translational studies and clinical trials must incorporate detailed assessments of local bone quality, fracture risk, and functional outcomes, integrating these with traditional oncology endpoints like progression-free survival and overall survival for comprehensive evaluation.

### 4.5 Toward clinical translation: Precision delivery, safety, and platform integration

The clinical translation of PROTACs to orthopaedics is fundamentally impeded by a 'physicochemical mismatch': these 'Beyond Rule of 5′ compounds exhibit high molecular weight and polarity that restrict passive diffusion through dense skeletal matrices [20]. To bridge this gap, delivery strategies must transcend generic encapsulation to employ rational, tissue-specific designs. For the negatively charged matrix of articular cartilage and intervertebral discs, cationic functionalization of carriers or degraders can exploit the Donnan equilibrium effect to enhance deep-zone penetration [100]; conversely, for mineralized bone, 'bone-homing' conjugates utilizing bisphosphonates or acidic oligopeptides can act as molecular hooks to target hydroxyapatite surfaces [108]. Building on these principles, recent advances have showcased the demonstrated performance of orthopaedic-specific delivery vehicles. For instance, intra-articular sustained-release hydrogels have been engineered to provide localized, prolonged exposure of PROTACs within the joint cavity, effectively minimizing systemic leakage [72]. Furthermore, highly biocompatible bone-targeting nanocarriers and engineered nanovesicles have emerged as potent vehicles capable of deeply penetrating the dense extracellular matrix to deliver degraders directly to chondrocytes or synovial macrophages [109].Crucially, deep learning is poised to revolutionize this domain by shifting predictive focus from 'potency' to 'penetrability.' Future architectures, such as Graph Neural Networks trained on diffusion data, must aim to optimize the diffusivity (*D*) and partition coefficients of these degrader-carrier systems within collagen-apatite networks.Specifically, computational approaches can simulate and optimize critical biophysical parameters—such as the surface zeta potential, hydrodynamic size of nanocarriers, and the degradation kinetics of hydrogels—ensuring that biologically active candidates are physically capable of accessing their sequestered targets while maintaining sustained release profiles [110].

Beyond delivery itself, long-term safety and resistance mechanisms constitute core hurdles for clinical entry. The catalytic nature of TPD implies pharmacological effects that may outlast plasma half-life, posing risks of delayed adverse events in slow-turnover bone tissues. Consequently, traditional toxicity protocols must be adapted to skeletal specifics, incorporating longitudinal imaging, histomorphometry, and biomechanical testing to assess impacts on bone structural integrity.Furthermore, early-phase clinical trials must be fundamentally tailored to orthopaedic realities to validate target engagement in avascular niches. For instance, a Phase Ia trial for an intra-articular PROTAC targeting osteoarthritis or rheumatoid arthritis could employ a robust “window-of-opportunity” design. By administering the degrader to end-stage patients scheduled for total knee arthroplasty (TKA) several weeks prior to surgery, researchers can overcome the traditional “black box” of joint pharmacokinetics. The subsequent retrieval of synovial fluid, excised synovium, and resected cartilage during TKA allows for spatial mass spectrometry imaging (MSI) and quantitative proteomics. This approach provides definitive, direct evidence of whether the PROTAC successfully penetrated the dense cartilage extracellular matrix or effectively reprogrammed target proteins within synovial macrophages, rather than relying solely on systemic serum biomarkers.

Concurrently, evaluating early clinical efficacy requires a paradigm shift in imaging. Traditional Dual-Energy X-ray Absorptiometry (DXA) measures two-dimensional areal bone mineral density, rendering it insufficient for detecting the nuanced, early-stage microenvironmental shifts induced by TPD. Future trials must prioritize highly sensitive, three-dimensional imaging biomarkers. High-Resolution Peripheral Quantitative Computed Tomography (HR-pQCT) is essential for dynamically monitoring early therapeutic changes in trabecular microarchitecture and cortical porosity [111]. Similarly, T2 and T1ρ mapping MRI are critical for detecting subtle restorations in cartilage matrix composition, such as proteoglycan and collagen network integrity [112]. Crucially, when these advanced imaging modalities are coupled with deep learning-based radiomics, algorithms can detect sub-visual textural features and predict long-term structural tissue preservation, providing highly sensitive, non-invasive readouts for orthopaedic TPD efficacy.Deep learning models analyzing longitudinal multi-omics data offer a powerful adjunct in this context, capable of dynamically identifying emerging resistance signatures or safety signals to inform the iterative optimization of dosing regimens and target-E3 pairings.

Finally, scalable advancement relies on establishing a platform-level infrastructure that seamlessly integrates data, algorithms, and experimentation. A closed-loop pipeline—integrating multimodal data acquisition, AI-driven modeling, and high-throughput validation—is essential to perpetually update 'target-E3-disease' atlases. By integrating clinical registry data back into preclinical design, this learning-enabled cycle will elevate orthopaedic TPD from isolated case studies to a systematic therapeutic paradigm, necessitating deep collaboration across computational, biological, and clinical domains to realize the vision of precision microenvironmental reprogramming.

## 5 Outlook and future perspectives

Targeted protein degradation (TPD) has matured into a transformative therapeutic modality, offering a mechanistic approach to address previously undruggable targets in orthopaedics [113,114]. By co-opting the cell's endogenous quality control machineries—primarily the ubiquitin–proteasome system (UPS) and the autophagy–lysosome pathway—degraders catalytically eliminate pathogenic proteins driving aberrant bone and joint remodeling [115]. This strategy expands the druggable proteome to include refractory transcription factors, non-enzymatic scaffolds, and phase-separated signalling hubs that resist traditional occupancy-based inhibition [20,115]. When integrated with a systems-level understanding of skeletal biology—including the distinct physiology of osteocytes, chondrocytes, and synovial fibroblasts, as well as the anatomical constraints of the avascular, mineralized niche—TPD provides a framework to shift the clinical paradigm. The field is now positioned to move beyond palliative symptom management and structural replacement toward mechanism-based microenvironmental reprogramming. However, to fully realize this transition, the field must explicitly confront the current translational gap. While systemic degraders such as bavdegalutamide (ARV-110) and vepdegestrant (ARV-471) have rapidly advanced into late-stage clinical trials for oncology, providing tangible survival benefits, TPD applications for non-malignant orthopaedic conditions remain largely confined to *in vitro* studies and early animal models [106,107]. This stark contrast between the rapid clinical progress in oncology and the nascent state of orthopaedic TPD highlights a critical bottleneck: the severe physicochemical mismatch between conventional degrader molecules and the avascular, dense skeletal matrix. Overcoming this barrier underscores an urgent “call to action"—we must leverage artificial intelligence not merely for target discovery, but to prioritize tissue penetrability from the very inception of drug design.The concurrent rise of deep learning and multimodal data integration serves as a catalyst for this transition, offering an opportunity to rationalize orthopaedic TPD development beyond the inefficiencies of empirical screening [45,64].

In this data-driven era, computational architectures are increasingly being explored to support multiple stages of the discovery pipeline. Protein language models and geometric deep learning networks (e.g., SE(3)-equivariant graph neural networks) have shown promise in predicting protein–protein interactions and may assist in prioritizing degradable targets within skeletal disease networks, although their application to orthopaedic TPD remains nascent [62,116,117].Generative AI models, including diffusion-based architectures, are also being investigated for the *de novo* design of PROTACss and molecular glues; early proof-of-concept studies suggest potential to optimize ternary complex stability, cooperativity, and physicochemical properties, yet experimental validation in orthopaedic contexts is still limited [64]. Simultaneously, the integration of single-cell spatial transcriptomics, structural biology, and clinical radiomics offers a conceptual pathway toward constructing cross-scale disease maps [81,[118], [119], [120]]. Such maps could, in principle, link molecular degradation events to macroscale clinical outcomes—such as cartilage regeneration or trabecular bone strengthening—moving ‘precision degradation’ from concept toward feasibility.Furthermore, to fully realize the vision of “microenvironmental reprogramming,” the convergence of AI and TPD must eventually transcend the ubiquitin-proteasome system. A substantial fraction of orthopaedic pathology is driven by extracellular factors (e.g., inflammatory cytokines, growth factors) and intracellular aggregates (e.g., misfolded collagen) that are resistant to proteasomal degradation [14]. Future computational frameworks may eventually need to expand to model lysosome-targeting chimeras (LYTACs) and autophagy-targeting chimeras (AUTACs). In principle, training geometric deep learning models on the interactions between degraders and lysosome-shuttling receptors (e.g., CI-M6PR) or autophagy adapters (e.g., LC3) could extend the reach of degradation therapies to the entire skeletal proteome—a goal that remains entirely conceptual at present.

It is imperative to offer a balanced assessment of the limitations inherent in both this review and the broader field. Because the intersection of deep learning and orthopaedic TPD is still in its infancy, a primary limitation of this current review is its reliance on extrapolating computational frameworks and success stories from oncology. The practical combination of TPD and computational technologies in orthopaedics faces massive hurdles, chief among them being the “small data” problem. Enhancing the quality and breadth of training datasets is essential, as the field currently lacks large-scale, standardized skeletal multi-omics and structural data (e.g., crystallized orthopaedic ternary complexes). To bridge this gap, researchers must deploy advanced domain adaptation and transfer learning techniques to leverage vast oncology data reservoirs [45,117].Furthermore, to facilitate clinical adoption, AI models must evolve from ‘black boxes’ toward greater mechanistic interpretability (explainable AI, XAI); clinicians need to understand exactly why a model predicts a specific target-E3 pair or toxicity risk. Biologically, the field must also move beyond static snapshots. Efforts to comprehensively map the ‘skeletal ubiquitinome’—delineating how E3 ligase activity fluctuates under physiological loading, aging, and inflammatory stress—are urgently needed to provide accurate contextual data for AI training [121,122].

From a translational perspective, the unique anatomical barriers of the skeletal system represent the ultimate final-mile obstacle. *In silico* success does not guarantee in vivo penetration. The development of innovative, spatially controlled delivery systems—capable of navigating avascular cartilage or mineralized bone matrix—remains a critical enabler [[123], [124], [125], [126], [127]].Concurrently, rigorous long-term safety monitoring integrating multi-omics is essential to detect delayed skeletal toxicity in long-lived cells like osteocytes [113,119,120].To systematically address these limitations and make the integration of computational methods, TPD, and orthopaedic medicine more actionable, we propose a clear 5-to-10-year developmental roadmap identifying key milestones and research tasks:

**Phase I (Years 1**–**3): Data Generation and AI Calibration**. The primary task is to resolve the “small data” bottleneck. Key milestones include the construction of joint-specific spatial transcriptomic atlases and the comprehensive mapping of the skeletal ubiquitinome. Computationally, generative AI must be calibrated specifically for skeletal targets using these new datasets.

**Phase II (Years 3**–**5): Precision Delivery and Preclinical Validation**. Research must shift toward coupling AI-optimized degraders with orthopaedic-specific delivery vehicles (e.g., bone-homing nanocarriers, intra-articular hydrogels). A critical milestone is validating their matrix penetrability and safety in sophisticated next-generation models, such as microphysiological ‘organ-on-a-chip’ platforms and patient-derived organoids that faithfully recapitulate human biomechanical loading and matrix stiffness [[128], [129], [130]].

**Phase III (Years 5**–**10): Clinical Translation and Closed-Loop Refinement**. The ultimate milestone is the initiation of biomarker-stratified Phase Ia “window-of-opportunity” clinical trials. Main tasks involve utilizing advanced AI-radiomics (e.g., HR-pQCT) and spatial proteomics to dynamically monitor deep-tissue target knockdown and structural safety in patients, thereby establishing a closed-loop framework that feeds clinical registry data back into preclinical AI design.

Notwithstanding these significant hurdles, the strategic convergence of TPD with AI-driven skeletal research presents a transformative opportunity. This convergence offers a chance to reframe conditions like osteoarthritis and osteoporosis not as inevitable consequences of aging and mechanical wear, but as pathologies amenable to targeted molecular intervention. If successfully realized, a deep learning-enabled TPD platform could unlock novel therapeutic windows across the spectrum of orthopaedics—from degenerative joint diseases and intervertebral disc degeneration to malignant bone pathologies. Such an outcome would represent not merely an incremental advance in drug discovery, but a fundamental shift in clinical focus: from palliation and structural replacement toward mechanism-based tissue repair and restoration.

## Ethical statement

This is a review article. No animal or human studies were carried out by the authors for this article; therefore, ethical approval was not required.

## Data availability statement

Data sharing is not applicable to this article as no new data were created or analyzed in this study.

## Author contributions

Jianxiong Ma: Conceptualization, Supervision, Project administration, Funding acquisition, Writing – review & editing. Lulu Zhang: Conceptualization, Methodology, Investigation, Writing – original draft, Visualization. Yan Wang: Resources, Visualization, Writing – review & editing. Dong Wang: Resources, Validation, Writing – review & editing.

## Funding

This work was supported by the 10.13039/501100001809National Natural Science Foundation of China (NSFC) [Grant Number 82405109]; the Tianjin Education Commission Scientific Research Program [Grant Number 2024ZXZD001]; the Tianjin Science and Technology Plan Project [Grant Number 25JCZDJC01350]; the Key Project of Tianjin Science and Technology Major Project and Engineering Public Health Project [Grant Number 25ZXWZSY00010]; and the Open Fund of the Key Laboratory of Emergency Management Department for Key Technologies and Equipment of Medical Rescue [Grant Number YJBKFKT202508].

## Declaration of competing interest

The authors declare that they have no known competing financial interests or personal relationships that could have appeared to influence the work reported in this paper.
